# An Audit of Risk Acknowledgment of Valproate Use in Women (General Adult Psychiatry)

**DOI:** 10.1192/bjo.2022.456

**Published:** 2022-06-20

**Authors:** Elham Khattab, Priti Singh

**Affiliations:** NHS Grampian, Aberdeen, United Kingdom

## Abstract

**Aims:**

- To enable identification, recall of all women (18–60 years) who may be of childbearing potential and are currently prescribed sodium valproate and identify those that are at risk.
- To check that the patients continue to meet the conditions of the Pregnancy Prevention Programme.
- To ensure that the guidelines by MHRA are adequately followed.
- To evaluate our practice relating to completing the Risk Acknowledgement Form for Sodium Valproate.

**Methods:**

**First audit cycle August 2021:**

From the General Adult Database (NHS GRAMPIAN), we identified 33 women between the ages of 18–60 years who were prescribed Sodium Valproate as a mood stabiliser in the period between August 2020 until August 2021. Data were obtained from patients’ records to ensure patients were still open to psychiatry services, compliant with Sodium Valproate, had regular contact with specialists and identified Valproate risk acknowledge form existed and adequately filled.

Univariate analysis was used to analyze the result.

THE INCLUSION CRITERIA:

- Adult female patients(18–60) who are open to psychiatry services.
- On Sodium Valproate as a mood stabilizer.

**Second audit February 2022:**

A second audit was conducted using the same standards and timescale as for the primary audit. Using telephone and emails, the teams were contacted and encouraged to complete the relevant documentation.

**Results:**

**First audit cycle August 2021:**

- There were a total of 33 patients included in the audit.
- 97% of the patients were in contact with psychiatry services and specialists.
- **Only one patient had an Annual Risk Acknowledgement Form (ARAF) filled and scanned to her E-notes.**
- 66% of women were between the ages of 45–60 years of age.

**Second audit cycle February 2022**

The results showed 39 female patients (18–60) were on Sodium Valproate as a mood stabiliser. The mean age was 45 (18–60). We identified Completed Annual Risk Acknowledgement Form (ARAF) forms on 21 patients.

**The proportion of completed ARAF was increased from 3% to 54%.**

**Conclusion:**

**Conclusion of the first cycle:**

97% of the patients were in contact with psychiatry services and specialists.

**• Only one patient had an Annual Risk Acknowledgement Form (ARAF) filled and scanned to her E-notes.**

**Conclusion of the second cycle:**

There was a significant increase in compliance with the MHRA guidelines regarding Sodium Valproate prescription in women of childbearing age in our department.

**The proportion of completed ARAF was increased from 3% to 54%.**

- Valproate is highly teratogenic, and evidence supports that use in pregnancy leads to neurodevelopmental disorders (approx. 30– 40% risk) and congenital malformations (approx. 10% risk).
- Valproate must not be used in women and girls of childbearing potential unless the conditions of the Pregnancy Prevention Programme are met and only if other treatments are ineffective or not tolerated, as judged by an experienced specialist.
- The MHRA advises that all healthcare professionals must continue to identify and review all female patients on valproate.
- The Annual Risk Acknowledgement Form should be used for all future reviews of female patients on valproate
- Specialists should comply with guidance given on the form if they consider the patient is not at risk of pregnancy, including the need for review in case her risk status changes.

